# Retraction: Matteucci, E., et al. Microenvironment Stimuli HGF and Hypoxia Differently Affected miR-125b and Ets-1 Function with Opposite Effects on the Invasiveness of Bone Metastatic Cells: A Comparison with Breast Carcinoma Cells. *Int. J. Mol. Sci.* 2018, *19*, 258

**DOI:** 10.3390/ijms21010154

**Published:** 2019-12-25

**Authors:** International Journal of Molecular Sciences Editorial Office

**Affiliations:** MDPI, St. Alban-Anlage 66, 4052 Basel, Switzerland; ijms@mdpi.com

We have been made aware that a number of figures of the title paper [1] contains unacceptable manipulations and modifications. In particular, Figure 3D contains duplicated points and in Figures 1D and 5B parts of the background have been duplicated. The authors have not been able to provide a satisfactory explanation for these irregularities and the journal Editors no longer have confidence in the conclusions of the paper. Therefore, to ensure the addition of only high-quality scientific works to the field of scholarly publication, this paper [1] is retracted and shall be marked accordingly. MDPI is a member of the Committee on Publication Ethics and takes responsibility to enforce strict ethical policies and standards very seriously. We apologize to our readership that this went undetected until now.
